# Chronic illness and financial burden in Switzerland (FINK): a protocol for a mixed methods research project

**DOI:** 10.1136/bmjopen-2024-089743

**Published:** 2024-11-19

**Authors:** Karin Ribi, Andrea Schöpf-Lazzarino, Rosa M.S. Visscher, Josip Jurisic, Elena Alder, Bettina Schwind

**Affiliations:** 1 Careum School of Health, part of the Kalaidos University of Applied Sciences, Zurich, Switzerland; 2 University of Zurich Institute of Biomedical Ethics History of Medicine, Zurich, ZH, Switzerland

**Keywords:** Chronic Disease, Caregiver Burden, Quality of Life

## Abstract

**Abstract:**

**Introduction:**

People with cancer and other chronic illnesses, their families and caregivers report financial burden as a problem that affects multiple aspects of daily life. While cancer research has coined the term ‘financial toxicity’ to describe the financial consequences, the understanding, development and operationalisation of the concept of financial burden are still incomplete, particularly regarding subjective financial burden and its relationship with well-being. The overall aim is to investigate financial burden and its implications for the well-being of people living with chronic illness, their families, and caregivers in Switzerland.

**Methods and analysis:**

Transdisciplinary discussion groups and a participatory action design element frame: (1) a conceptualisation using a hybrid concept analysis approach and (2) an assessment of financial burden of chronic illness in relation to well-being. The hybrid concept analysis combines the results of a scoping review with a secondary analysis of existing interviews using computational linguistics and qualitative analysis. The assessment phase will investigate the extent and nature of financial burden through a nationwide survey. Results from mobile diaries/interviews will contribute to both—the conceptualisation and assessment phases.

**Ethics and dissemination:**

The Ethics Committee of the Canton Zurich, Switzerland, did not consider the research project to fall under the Swiss Law on Human Subjects research and issued a waiver (Req-2O23-01496). The project respects all the rules and regulations in the Swiss Federal Act on Data Protection and those by the Swiss Federal Official Responsible for Data Protection and Transparency. Results will be disseminated through presentations at conferences and publications in peer-reviewed journals and through the established multi-stakeholder network.

**STRENGTHS AND LIMITATIONS OF THIS STUDY**

- To our knowledge, FINK is the first research project to address the financial burden of different chronic illnesses using a transdisciplinary and participatory research approach.
- Transdisciplinary and participatory research approaches ensure the practical relevance of the results but require an openness and flexibility that can make the research process challenging within given funding limits.
- In spite of the novelty of the methodology of the FINK project, the data will be specific to Switzerland, and their transferability to other countries will be limited.
- Recruiting participants through various health and social care stakeholders has the advantage of reaching those most in need, but the data may not be representative.
- The FINK project will provide not only solid quantitative and qualitative data, but also key insights and solutions that are considered important to change current policies and practices that have stigmatised financial burden in affluent Switzerland.

## Introduction

 Healthcare costs are rising worldwide.^1^ In Switzerland, about 80% of the direct healthcare costs can be attributed to chronic illnesses, half of which are caused by treating the five most prevalent groups, that is, cancer, diabetes, musculoskeletal disorders, and cardiovascular and chronic respiratory diseases.^2^ Switzerland has also one of the highest per capita out-of-pocket costs (OOPC) among 10 high-income countries.^3^ The share financed by private households accounted for the majority of health costs (64.9%) in 2019^4^ and is steadily increasing causing concerns also for middle class families.^5^

Most research about individual financial aspects of chronic illness originated in the USA with a focus on cancer and coined the terms ‘financial toxicity’, ‘financial burden’, ‘financial hardship’ or ‘financial distress’.^6^,^8^ Systematic reviews indicate that 45–49% of patients with cancer experience financial burden.^7 9 10^ Outside of the USA, the prevalence of financial distress in cancer patients ranges from 16% to 73%,^10^ while in publicly funded healthcare systems, the financial burden was less prevalent, with 22–27% of cancer patients reporting to be affected.^11^ Individual financial burden was also detected in studies focusing on chronic diseases other than cancer.^12^,^15^ Financial burden of living with chronic illnesses was negatively associated with health-related quality of life^712 15^,^17^ and can also lead to treatment non-adherence and delaying or forgoing medical care as shown for different chronic illnesses.^713 18^,^20^ Evidence also indicates financial implications for families and caregivers of patients with chronic illnesses such as opportunity costs of informal care time and caregiver time loss from paid employment.^1521^,^24^ Changes in roles and responsibilities or relationships, decreased social life, changes in the living arrangement or a family member work adjustments are reported to affect family dynamics,^25^,^27^ and lead to lower quality of life in patients and caregivers.^28^

Several conceptual models or frameworks of financial burden have been developed.^6 8 9 11 12 29^ They differ in terms of number and type of domains or aspects included. However, two main domains can be distinguished irrespective of chronic illness:^8 12 15 29^ (1) the objective financial burden due to illness covers direct medical and non-medical (eg, OOPC or travel and accommodation costs) and indirect costs (eg, income loss for patients and caregivers) and (2) the subjective financial burden that includes three sub-dimensions—(1) the subjective perception of the material condition (eg, OOPC, missed work), (2) the psychosocial reaction to the financial burden (eg, feeling distressed or worry) and (3) behavioural reactions or coping strategies to capture behaviour changes (eg, reduced medication adherence, delayed/forgone care).

At present, research about individual financial aspects of chronic illness is limited in several ways. The concept’s understanding, development and operationalisation are still incomplete, particularly regarding subjective financial burden and its relationship with well-being. Available conceptual models are predominately based on results from quantitative approaches (eg, questionnaires). Evidence from qualitative studies indicates that subjective financial burden is more multifaceted. Living through the experience of financial burden is rather complex, circular and variable.^2730^,^32^ Subjective financial burden does not necessarily have to be a consequence of objective financial burden; but it can arise from the anticipation of potential problems such as worsening health or job loss.^33^ Existing concepts are primarily disease-specific, focusing on the individual patient rather than the implications for the daily lives of ill persons, their families, and caregivers or structural factors of a country’s social welfare system. Thus, a better understanding of the complex relationships between financial burden, well-being and the social context of individuals with chronic illness is needed. This is important to empower professionals from the health and social sector to provide interprofessional, coordinated and sustainable support to all affected by financial burden due to chronic illness.^34^

The overall objective of this research project is to investigate financial burden and its implications for the well-being of people living with chronic illness, their families and caregivers in Switzerland. We focus on the five most common chronic disease groups in Switzerland (cancer, diabetes, musculoskeletal disorders, and cardiovascular and chronic respiratory diseases). Specific aims are: (1) to conceptualise financial burden and its implications for the well-being of people with chronic illness, their families and caregivers, and (2. to assess the extent of financial burden and its implications.

## Methods and analysis

**Figure 1:**
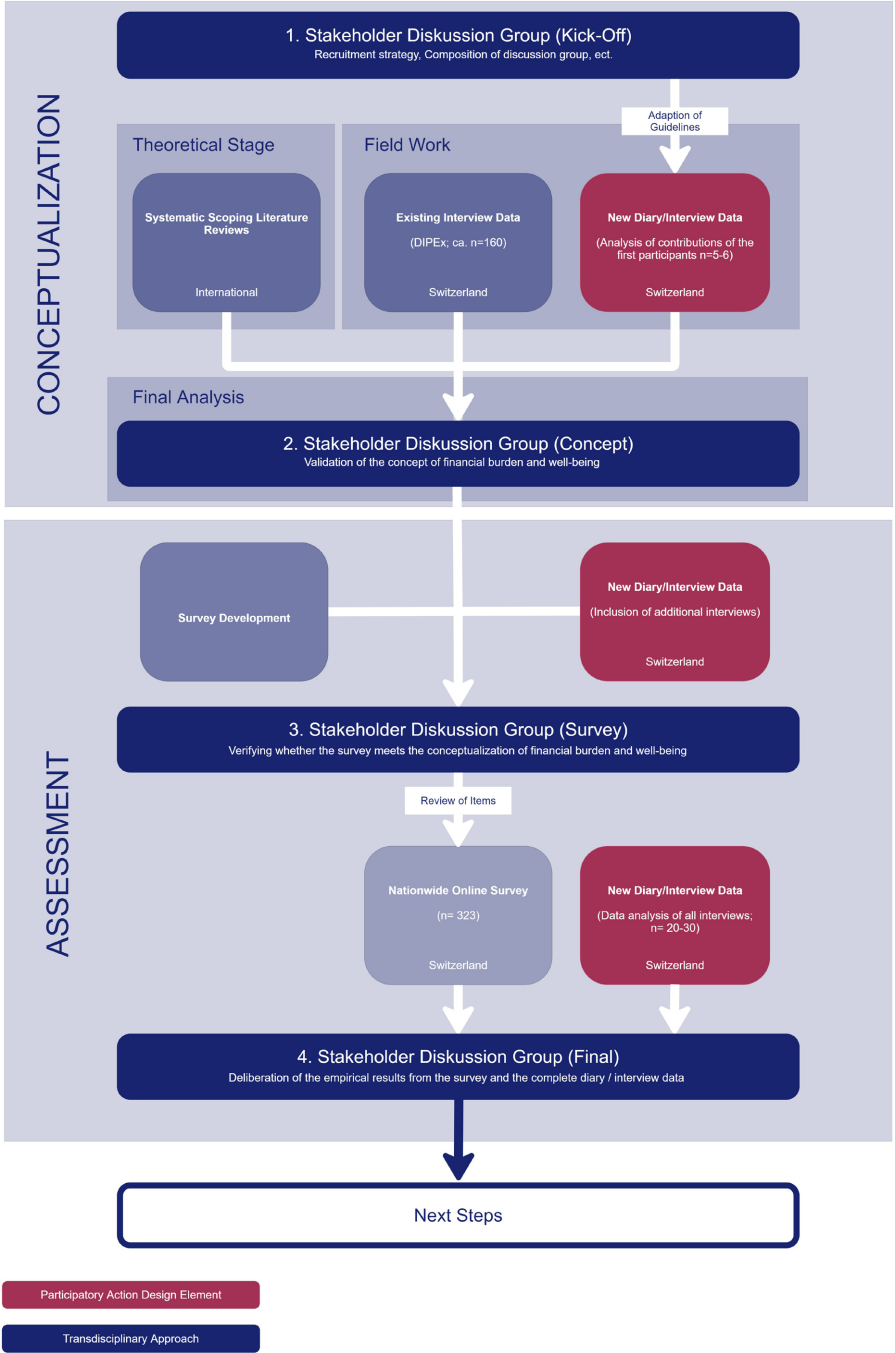
Overview of the mixed-methods research process.

The research project started in January 2024 and will be completed by the end of March 2027. It is divided into two sequential intersecting phases: (1) conceptualisation and (2) assessment. Each phase is characterised by convergent mixed-methods research activities.^35^ The two phases are framed by a transdisciplinary approach and a participatory action design element (figure 1). The transdisciplinary approach aims to ensure that the project is relevant to the realities of the Swiss context and accessible to social and healthcare scientists, practitioners and policymakers. The participatory action design element aims to give voice to individuals affected by financial marginalisation and social inequity.^35 36^ Including the ‘less heard’ is warranted, as poverty issues in a wealthy country like Switzerland may be considered or experienced as taboo.^37^

The research project is funded by the Swiss National Science Foundation which launched a funding scheme in 2023 to promote research on ‘Health and Well-being’ at universities of applied sciences and teacher education (10HW-E_220691). To receive such a positive funding decision, a SNF peer-review process had to be passed successfully. For more details, see https://data.snf.ch/grants/grant/220691.

### Conceptualisation

The aim of phase 1 is to create a concise concept of financial burden for the Swiss context. This is achieved by following the hybrid concept analysis approach used in nursing science to understand the ambiguities of concepts and evaluate them in their contexts.^38 39^ It comprises three stages—a theoretical stage, fieldwork, and the final analysis—and allows for the combination of deductive and inductive approaches.

#### Theoretical stage

The aim of the theoretical stage is to develop a preliminary concept based on the international scientific literature on chronic illness and financial burden. We will conduct a systematic scoping literature search^40^ to find existing concepts of subjective financial burden in relation to well-being developed for (specific) chronic illnesses and to evaluate major points of contrast and similarity.^38^ This allows for the integration of disciplines such as nursing, medicine, psychology, sociology, social work, public health and philosophy (databases: Medline, CINAHL, Embase, Cochrane Reviews & Trials, PsycINFO, EconLit). The review decision process will be detailed following the PRISMA extension for Scoping Reviews. A librarian from the medical library, University of Zurich, will conduct the literature search. At least two researchers with different disciplinary backgrounds will perform the screening, selection and analysis to ensure the rigour and quality of the process and reduce interpretation bias. The scoping review protocol is available on Open Science Framework (https://osf.io/ajre6/)

#### Fieldwork

The fieldwork aims to refine the concept by integrating empirical elements that are important and specific to the Swiss context. Therefore, we will conduct a secondary analysis of existing qualitative interviews on aspects of living with a chronic illness in Switzerland. This data set comprises more than 160 narrative interviews with patients with dementia, multiple sclerosis, chronic pain, Parkinson’s disease and rare diseases. It was collected by the DIPEx (Database for Individual Patient Experiences, www.dipex.ch) research group at the Institute of Biomedical Ethics and History of Medicine, University Zurich. Although not comprising the five most prevalent chronic illnesses in Switzerland, we expect to get relevant insights on subjective financial burden of people with chronic illnesses specific to the Swiss context. The analysis of the DIPEx interviews will combine natural language processing based on a lemma/named entity analysis, sentiment analysis, distributional semantics and rule-based coding^41^,^43^ with in-depth qualitative analysis. This strategy is best suited to effectively search large amounts of texts in a timely yet structured manner to conduct qualitative analysis thereafter to obtain an in-depth understanding of the matter.^44^ We will also conduct new in-depth diaries/interviews (n=5–6) as part of the participatory action element for the five most relevant chronic illnesses (see corresponding section for details).

#### Final analysis

The aim of the final analysis is to see how the preliminary concept based on the scoping review does compare and contrast with the results from the DIPEx interviews and the diaries/interviews. The applicability of the concept for the Swiss health and social context will be discussed and validated with stakeholders (see second stakeholder discussion group).

### Assessment

The aim of phase 2 is to assess the extent and nature of financial burden and its implications for the well-being of people with chronic illness, their families and caregivers in Switzerland using a national survey. The results of the survey will be complemented by the results of the mobile phone diaries and qualitative in-depth interviews as part of the action design element.

#### Development of survey

Based on the results from the conceptual phase, a cross-sectional, nationwide survey in three languages (German, French and Italian) will be developed. Survey data will likely consist of a combination of existing validated instruments and ad hoc questions (for research project purposes) to ensure that all aspects of the concept are covered. Results from the first diaries and interviews will also be considered for the development of survey questions. Potential instruments that are considered relevant in this context assess financial toxicity (eg, COmprehensive Score for Financial Toxicity^45 46^), family or caregivers burden (eg, Burden Scale for Family Caregivers^47^), and well-being (eg, WHO Quality of Life Brief Version (WHOQOL-BREF), the WHOQOL Group,^48^ European Quality of Life 5 Dimensions 5 Level Version,^49^ Patient-Reported Outcomes Measurement Information System-29,^50^ WHO-5 Wellbeing Index,^51^ ICEpop CAPability measure for Adults^52^). Socio-demographic data will be collected through standardised questions informed by Swiss health survey.

#### Sample

We aim to target people who are affected by at least one of the five most common chronic illness groups, their family and caregivers. Participants are required to be at least 18 years old and knowledgeable in German, French or Italian. To estimate the required sample size for the survey, the formula for the prevalence of a qualitative variable was used.^53^ The expected proportion of people experiencing financial burden was set at 30% based on the available evidence across chronic illnesses,^11 14 15 33^ with a 95% CI (z=1.96, and the absolute error set to 5%) requiring a sample size of 323 participants. We expect a response rate of 30%,^54^ which will require to contact up to 1000 chronically ill persons, their family members and caregivers.

#### Recruitment and data collection

Participants will be recruited via institutions that are represented through the stakeholder’s who will be involved in the transdisciplinary part of the project, especially patient organisations. The online survey will be carried out over 6 to 8 months using a web-based survey tool (Unipark, Tivian XI GmbH, Germany). Supporting institutions will send a link to the survey via email to their members and/or publish the link on newsletters, websites, social media posts, and/or through flyers/posters at local medical and social institutions. Participants can decide if they want to enter a raffle. The survey will be set up in a way that the main answers and email addresses (if provided by participants for the raffle) are saved separately. Only the survey coordinator will be able to access the email addresses and contact the winners of the raffle. Any data entry from hard copy or recording to electronic text or tabular data will be checked by at least two designated project team members for quality assurance.

#### Analysis

Descriptive statistics will be used to assess the proportion of chronically ill persons that experience financial burden. To identify possible risk factors, the effect of socio-demographic factors on financial burden outcomes will be investigated through linear regressions. Correlation analysis will be conducted to estimate the relation between financial burden and well-being for persons with chronic illness, their families and caregivers. Data will be analysed with SPSS v25 (SPSS Inc., IBM, IL USA). Depending on the final survey set-up and obtained data sample, the exact analysis proceedings will be defined in collaboration with the stakeholders (see third stakeholder discussion group) and a statistician.

### Participatory action design element

This part combines mobile phone diaries with follow-up qualitative in-depth interviewing, applying the photo-elicitation technique.^55 56^ This approach is situated within the realms of qualitative participatory research and integrates innovative digital elements rooted in participatory action design,^56 57^ best suited for tracing changes over time, making implicit daily practices and prerequisite knowledge explicit and tangible.^58^

#### Development of diaries and interview development

For the diaries, we will develop a short guide comprising prompts regarding (1) experiences with their current financial situation, (2) the consequences for themselves and (3) others, (4) applied coping strategies and (5) support received. At the end of the diary period, an elicitation interview will be conducted, in which diary entries (as in printed copies of photos, texts and/or transcripts of audio recordings) are reflected to understand their relevance, contextual aspects, and circumstances to participants’ lives, financial burden and well-being. This approach lends itself to maximising participants reflexivity and recall.^56^

#### Sample

In addition to the criteria for the survey participants, diary/interview participants must have a history of (self-perceived) financial burden due to chronic illness, have a smartphone, and be willing to instal and use the messenger service Threema. Samples are drawn from across the three main language regions of Switzerland. To ensure maximum variety in sampling, the criteria are at least one of the five most common chronic disease groups in Switzerland, different ages, gender and household status (members per household). We plan to include 20 to 30 chronically ill persons, family members and caregivers.

#### Recruitment and data collection

Like the survey participants, diary/interview participants will be recruited via supporting stakeholders. Depending on the opportunities, possibilities and preferences of stakeholders, information about the research project is published on websites or newsletters of stakeholders, or eligible persons are approached by their staff. Data collection will start with an onboarding, in which participants will be trained in keeping mobile phone diaries and the ethical aspects related to how to use cameras and what (not) to capture.^56^ The onboarding conversation will be face-to-face or online and will not be recorded. Thereafter, participants are asked to complete a 2-to-3-month mobile phone diary (photo, audio, or written diary and/or a combination thereof) using the messenger service Threema. Participants are asked to create at least one entry per week. Finally, participants will be invited to participate in the elicitation interview. The interview is conducted face-to-face or online and recorded. In recognition of this valuable and personal contribution, compensation will be provided in the form of a voucher.

Each diary/interview participant will be assigned a unique number that is not linked to participant-identifiable information. The identification key linking the participant’s unique number and personal information will be stored separately from the data on an encrypted universal serial bus stick or external hard drive, which will be stored in a secure desk pedestal, accessible only to the research team or nominated representatives. It is necessary to retain this information to be able to remove the data if the research project is discontinued. All audio-recorded diary entries and elicitation interviews are transcribed and pseudo-anonymised promptly after collection. The raw data will then be deleted immediately. A professional transcription service will be used that offers encrypted data transmission and requires their employees to sign a confidentiality agreement.

#### Analysis

All textual data are analysed following reflexive thematic analysis as detailed by Braun and Clarke.^59^ For interactions between visual and textual data, textual visual-thematic analysis is applied.^60^ The analysis is supported by using MAXQDA (VERBI Software GmbH, Germany). The analysis will be done by project members with various disciplinary backgrounds, and the results will be discussed with the stakeholder discussion group.

### Transdisciplinary approach

Throughout the research project, we will conduct four stakeholder discussion groups to ensure that all activities and the applied methods are discussed and approved by the stakeholders from research, policy and practice.

The *first* discussion group (kick-off) aims to get insight on stakeholders’ real-life experiences with the topic. They can discuss dimensions, experiences and gaps regarding financial burden and well-being based on their own work, life and/or illness experiences. This input will be used to refine the diary and interview guides.

The *second* discussion group (concept) serves to finalise the conceptualisation of financial burden and well-being. Based on the results of the hybrid concept analysis, the discussion focuses on the joint identification of domains and aspects of financial burden across chronic illnesses and its implication for well-being on the individual and family levels.

The *third* discussion group (survey) will review and discuss the survey items and verify whether it meets the conceptualisation of financial burden and well-being.

The *fourth* discussion group (final) will deliberate the empirical results from the survey and the complete diary/interview data and identify next steps for research, policy and practice.

#### Sample

Each group will comprise about 15 stakeholders from research, practice and policy including members from patient organisations to integrate an adequate diversity in expertise and practice to shape the process. We follow a mix of a closed and open group structure: the closed structure consists of a core team of five to eight people, which may also serve as an advisory board when needed. The open group structure includes additional stakeholders depending on the steps and complexity of the transdisciplinary process. Diarists will be invited to contribute with their lived experience from the second discussion group onwards, if they feel comfortable doing so. Participants will be compensated by a lump sum.

#### Recruitment and data collection

During the application phase, we contacted various stakeholders from health research, patient organisations, healthcare providers, social work, health insurance and government organisations to ask for support. At the beginning of the project, which started in January 2024, we approached further stakeholders to achieve a balanced composition of the discussion groups, for example, regarding representation of all three language regions and illness groups. Missing perspectives might be identified during the first stakeholder discussion group and relevant stakeholders invited. Results of discussions will be collected by means of visual and textual data (eg, maps and minutes.) The stakeholder discussion groups will be moderated by a professional facilitator trained in design thinking. Design thinking allows addressing complex problems and has the potential to envision alternative futures for health and social care through new forms of innovation by valuing diversity and collaboration across disciplines, viewpoints and backgrounds.^61^

#### Analysis and integration

The findings from the conceptualisation and the assessment phases as well as the results from the action design element will be convergently triangulated to inform the stakeholder discussion groups as an important element to link the different methods used in the project and ensure the usefulness of approaches and results for social and healthcare scientists, practitioners and policymakers.

### Patient and public involvement

The transdisciplinary approach integrates the non-academic perspective through the involvement of different stakeholders across the health and social sector and provides the opportunity to influence the conduct of the project. Although stakeholders were not involved in the design of the project, the transdisciplinary and participatory processes require an openness for the research process and methods. Diary/interview participants are invited to join the stakeholder discussion groups to involve the less heard and to provide room for their own interpretations of their lived experiences. Based on their priorities, preferences and experiences, possible adaptations may be required during the project. This will ensure that the results are relevant for providing support for persons affected by financial burden due to chronic illnesses.^35^ The final discussion group will identify the next steps for innovative solutions around financial burden and well-being in Switzerland, discuss the plans for dissemination, and highlight further research gaps and the involvement of patients and their caregivers as well as other relevant stakeholders.

### Ethics and dissemination

The research project was submitted to the Ethics Committee of the Canton Zurich, Switzerland. The committee did not consider FINK to fall under the Swiss Law on Human Subjects research and issued a waiver (Req-2O23-01496). Informed consent procedures vary for the different elements of the research project. For the DIPEx data, the original informed consent comprised the re-use of data in subsequent research projects. Potential participants of the survey will be taken to an information sheet and will be asked to give their electronic informed consent before starting the survey. For the diaries/interviews, two separate consent forms will be signed: first and prior to data collection, all diary/interview participants will give written informed consent to allow collection and analysis of data for research purposes. Second and after the interview, participants are asked to sign a consent form to allow the use of data for other purposes related to further research and teaching. If a participant withdraws from the first consent after having participated in the diary/interview study, all diary entries, recordings and transcriptions will be deleted but only until the data has been transferred to the MAXQDA software for data analysis. For the participants of the stakeholder discussion groups, no informed consent is obtained, as we do not collect individual data on health but are interested in transdisciplinary group processes and outcomes. Therefore, participants will be asked for their verbal consent to keep the content of the discussions confidential.

All data (except audio recordings) will be stored for at least 10 years after the end of the project at the Careum School of Health. The signed informed consent forms from the diary/interview study will be destroyed after 10 years. Not-identifiable processed data sets will be shared through the Zenodo repository (Zenodo - Research. Shared). The non-identifiable data will be made available at the time of publication. All unpublished non-identifiable data will be deposited in the data repository within 12 months after publication. No personal (or identifiable) data of the participants will be published.

Results will be presented to academia at conferences and published in peer-reviewed journals. We expect that the co-created knowledge and insights developed by the project will be shared through the established stakeholder network according to their preferences (eg, newsletters, symposia, roundtable or webinar), thereby informing future directions of action, feeding into implementation and supporting priority setting. During the conduct, regular updates on the proceedings will be published via the project page on the website of Careum School of Health and LinkedIn.

## Discussion

The intended project will provide: (1) conceptualisation of financial burden relating to well-being in daily life for the Swiss context and (2) qualitative and quantitative data on financial burden and its implications on the chronically ill, their families and caregivers specific to Switzerland. In addition, we will establish a solid transdisciplinary network allowing us to jointly formulate and co-design the next steps to tackle key areas of concern in research, practice and policy.

The conduct of the research project is not without risks. Challenges in the recruitment of participants who will take part in the survey, complete the mobile diaries and participate in the interviews will be minimised with broad field access through the established networks with stakeholders. The drop-out of participants over time, because of a potentially high workload or declining interest due to the relatively low individual benefit, will be counteracted by establishing a researcher-participant relationship characterised by continuity, mutuality and trust.^62^ Sufficient time will be allocated to explain what is expected from the participant and for communication with participants during data collection. There is a possibility that those with the highest burden may not feel able to participate in this project. By incorporating the element of transdisciplinary and participatory action design, we seek to minimise the risk of missing potential aspects of financial burden/well-being and at-risk groups.

We expect that the co-created knowledge and insights will inform future directions of action, feeding into implementation and supporting priority setting. We seek to contribute to the overcoming of the rather fragmented support system in Switzerland, which may not sufficiently reach the chronically ill, families and caregivers who are most in need. We also expect to contribute to health and social care services that become more participatory and focused on the whole context of people’s lives, a recommendation for future healthcare based on the Swiss National Science Foundation Program 74 ‘Smarter Health Care’.^63^ The participatory action element is key to this, as it allows the integration of individuals, their families and caregivers in vulnerable life situations because in a wealthy country like Switzerland, individual financial issues due to illness may be considered taboo.^37^

## Data Availability

Data sharing not applicable as no datasets generated and/or analysed for this study.
